# Structural Parameters Affecting Electrothermal Properties of Woolen Knitted Fabrics Integrated with Silver-Coated Yarns

**DOI:** 10.3390/polym11101709

**Published:** 2019-10-18

**Authors:** Kexia Sun, Su Liu, Hairu Long

**Affiliations:** 1 College of Textiles, Donghua University, 2999 North Renmin Road, Songjiang District, Shanghai 201620, China; 2170035@mail.dhu.edu.cn; 2 Engineering Research Center of Technical Textile, Ministry of Education, Shanghai 201620, China; hrlong@dhu.edu.cn

**Keywords:** electrothermal properties, resistance values, electrothermal knitted conductive fabric, knitted structures

## Abstract

Recently, more and more researchers have focused on electrical textiles that can provide or convert energy to facilitate people’s lives. Knitting conductive yarns into ordinary fabrics is a common way for electrical textiles to transmit heat or electrical signals to humans. This paper is aimed at studying the resistance values and temperatures of electrothermal knitted conductive fabric (EKCF) subjected to certain voltages over time. Six types of EKCFs with structural differences were fabricated using a computerized flat knitting machine with intarsia technology. Uniform samples 10 × 10 cm in size were made from wool, as were two different specifications of silver-coated conductive yarns. The wool yarn and one silver-coated yarn were mixed to knit a resistance area 2 × 2 cm in size in the center of the EKCF to observe heating behaviors. The experiment results showed that when the EKCFs were subjected to certain voltages over time, the resistance values of the resistance area increased over a short time and then gradually decreased, and the temperature gradually increased in the first 1000 s and tended toward stability after a certain period of time. The structural coefficient κ between different knitted structures (which predicted the thermal properties of different EKCFs subjected to different voltages) was analyzed. These results are of great significance for predicting the electrothermal performance of EKCFs with different knitted structures. On the basis of these results, an optimized knitted structure was selected as the best EKCF for wearable textiles, and the findings contribute to the field of technological and intelligent electrothermal garments and related products.

## 1. Introduction

Conductive textiles are traditional textiles that incorporate conductive fibers or other types of materials employing different technologies, and they are widely used in many fields [1,2], such as sensors [3,4,5,6,7,8], heaters [9,10,11], capacitors [12,13], and electromagnetic shielding fabrics [14]. In addition, knitting technology has become more and more popular in forming conductive textiles because the method produces a flexible structure and shape for the final product. Due to concerns about energy conversion, there is increasing research on heating electrothermal knitted conductive fabrics (EKCFs) that can translate electrical energy into heat without affecting the human wearing experience [15]. As resistance is one of the most important factors that influence the properties of conductive materials, most previous research has focused on the resistance properties of knitted conductive fabrics. Some researchers have extended their studies to focus on the properties of knitted conductive fabrics with different structures [16,17,18]. In our previous work, we also explored the influence of float-and-tuck stitches on the resistance properties of knitted fabrics. It was found that the resistance value will decrease dramatically in accordance with an increase in float or tuck elements. Some researchers have investigated the heating properties of conductive fabrics under different voltages [19,20,21]. On the basis of previous research, it has been deduced that the electrothermal properties of EKCF will change in accordance with variations in the knitted structures and applied voltage. It is theorized that resistance will change in accordance with the heating temperature; on the other hand, the heating temperature will be influenced by the change in resistance. It is a complicated issue.

Therefore, the aim of this paper was to investigate the principles of resistance values and the temperature of knitted conductive fabrics with different structures when subjected to different voltages. Two sets of experiments were conducted to determine the resistance values of each sample under different voltages and the resistance and temperature changes of samples under different voltages. Analytical calculations were carried out for a comparison to the experimental results. This study will contribute to research work on the use of knitted conductive fabrics for electrothermal garments.

## 2. Theoretical Analysis 

### 2.1. Input Power 

When power P at a certain voltage U is applied to knitted fabric, the resistance of the knit fabric is $R_{0}$, and the heat Q is generated after a certain period of time, *dt*. Their relationship is expressed with Equation (1):

(1) $$Q=P\times dt=\frac{U^{2}}{R_{0}}dt.$$

### 2.2. Heat Loss

Due to the difference in temperature between the fabric and the environment (ambient temperature), there is a loss of heat S during the heating process, which corresponds to the heat dissipation coefficient α and the changes in temperature ΔT and time *dt*, as expressed in Equation (2):

(2) $$S=\alpha \times \Delta T\times dt=\alpha \times \left(T-T_{0}\right)\times dt,$$

where *T* is the temperature of the fabric, and *T*_0_ is the temperature of the environment.

### 2.3. Equilibrium Temperature

At the beginning of the heating process, the rate that the fabric is heated is more rapid than the rate that heat dissipates, and therefore the temperature increases quickly. After a certain period of time, when both rates of heating are nearly the same, the temperature of the fabric will reach equilibrium and no longer increase. At this time, it will reach the equilibrium temperature $T_{s}$, which is shown by Equations (3) and (4):

(3) $$\frac{U^{2}}{R_{0}}=\alpha \times \left(T_{s}-T_{0}\right),$$

(4) $$T_{s}=T_{0}+\frac{U^{2}}{R\times \alpha }.$$

The resistance R of different structures of conductive fabric is related to κ (κ is the structural coefficient of knitted fabrics with different structures compared to knit fabric), and the resistance is also affected by the temperature. The fabric temperature is affected by the applied voltage *U*, which is determined by using Equation (5):

(5) $$R=\kappa \times R_{0}\left(U\right).$$

Substituting Equation (5) into Equation (4) gives

(6) $$T_{s}=T_{0}+\frac{U^{2}}{\kappa \times R_{0}\left(U\right)\times \alpha }.$$

As a result, the structural coefficient κ of the fabric and the P will influence the accuracy of the predicted steady-state temperature.

## 3. Experimental Procedures

### 3.1. Materials

**Figure 1:**
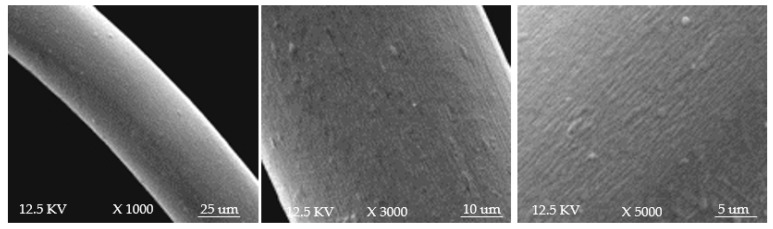
E-SEM micrographs of yarn B magnified 1000, 3000, and 5000 times from left to right (shot using a QUANTA environmental scanning electron microscope (FEI, Chech)).

Woolen yarn is widely used as a material for heating products due to its good thermal insulation effects. In this experiment, silver-coated yarns, woolen yarn, and polyester yarn were used to knit the conductive fabric. A coarse silver-coated yarn (yarn A) was used for the electrode part, a woolen and fine silver-coated yarn (yarn B) was used for the heating area, and a polyester yarn was used for nonconductive areas. In Figure 1, the SEM micrographs are images of yarn B magnified 1000, 3000, and 5000 times. Information on the specifications of the yarns is listed in Table 1. The electrode, heating area, and nonconductive areas were integrated by using a computer flat-knitting machine that applies intarsia technology.

### 3.2. Sample Preparation 

**Figure 2:**
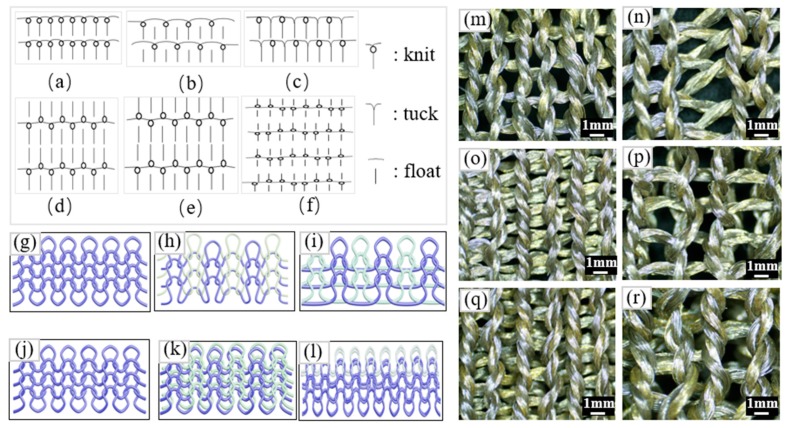
(**a**–**f**) The loop diagram, (**g**–**l**) simulation effects, and (**m**–**r**) fabric images of S1, S2, S3, S4, S5, and S6.

Samples were prepared with different knitted structures: three types of single-needle-bed knitted structures were numbered S1, S2, and S3 (knit, knit-and-float, and knit-and-tuck stitches), and three types of double-needle-bed knitted structures were numbered S4, S5, and S6 (1 × 1 rib, interlock, and derivative rib stitches). The loop diagrams, simulation effects, and fabric images of each sample are shown in Figure 2a–r, respectively. Three repeated samples of each structure were fabricated on an E12 Computerized flat knitting machine (Longxing, Jiangsu Jinlong Technology Co., Ltd., Changshu, China) (with corresponding knitting parameters listed in Table 2, where the NP value represents the length of the stitch, and the pulling value represents the pulling force of the roller on the fabric during knitting). The sample size was uniform at 10 cm × 10 cm, and the conductive region of the sample was uniform at 2 cm × 2 cm. Images of the samples with six different structures were taken by using a NIKON SMZ745T microscope (Tokyo, Japan) (magnified 30 times). Then, the samples were sealed and stored after being taken off of the machine without any washing treatment. 

### 3.3. Method

Experiments were conducted according to the fabric resistance test method (standard AATCC test method 76-1975). The experiment was carried out in a standard environment with a temperature of 20 °C, an atmospheric pressure, and a humidity of 65%. 

### 3.4. Experiment 1: Resistance Measurements of Samples

**Figure 3:**
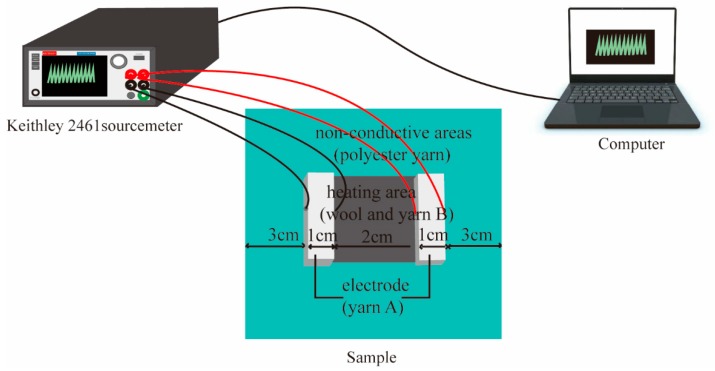
The tested device connections of the conductive fabric.

Resistance measurements of the samples were carried out by using a Keithley 2461 sourcemeter (Cleveland, Ohio, USA), which was interfaced with a computer, as shown in Figure 3. The electrode areas of the samples were covered by two electrode blocks with the same size to observe the changes in resistance values. External voltages of 1, 2, and 3 V were loaded onto the conductive areas through an adjustment of the computer. The resistance value of every sample was recorded on the computer after 60 s. Three samples of every structure were measured at each voltage, and the average value was calculated. 

### 3.5. Experiment 2: Simultaneous Testing of the Resistance and Temperature of Samples

Simultaneous testing of the resistance and temperature was conducted, and the instrument used for testing the temperature was a FLUKE TiS75 infrared camera (Everett, Washington, USA). Samples were placed on an insulating wooden board, and an infrared camera was placed 10 cm above the sample. Rechargeable batteries are usually made of nickel–hydrogen or nickel–chromium with a standard voltage of 1.2 V, and most heating products use portable rechargeable batteries. Considering the possibility of applying conductive samples to heating products, 1.2 and 2.4 V voltages were selected for the experiment for 1 h. Then, the resistance values were recorded by the computer at 1-s intervals. During the first 5 min (at 1, 2, 3, and 5 min) and then at every 5-min interval, the temperature was recorded to observe the variation of temperature with time.

## 4. Results and Discussion

### 4.1. Experiment 1

**Figure 4:**
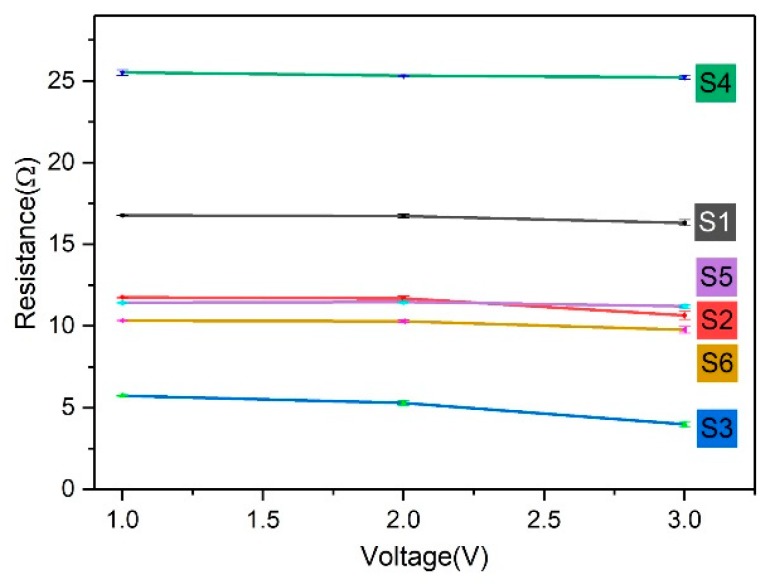
Resistance versus voltage curves of S1–S6 at 1, 2, and 3 V.

The resistance values of the samples with different structures at different voltages and resistance versus voltage curves are shown in Table 3 and Figure 4. The resistance values of three repeated samples were tested. It can be seen that the resistance values between different structures differed by nearly 20 Ω, and the resistance of the same structure decreased slightly (up to about 2 Ω) with an increase in applied voltage (1, 2, and 3 V). The electrical resistive network of the conductive fabric is a complex series and parallel circuit composed of two parts: the length-related resistance of the conductive yarn and the contact resistance generated by the contact between the yarns. We measured the length of yarn consumed to knit a 2 cm × 2 cm conductive area of different structures (S1: 146 cm; S2: 157 cm; S3: 199 cm; S4: 232 cm; S5: 273 cm; and S6: 306 cm). As can be seen in Table 3, the resistance values of the single-needle-bed structures at 1 V were S1 (16.77 Ω) (the largest), S2 (11.76 Ω) (the second largest), and S3 (5.74 Ω) (the smallest). This was contrary to the results considering only the length resistance value. Table 2 shows that the total number of stitches in the conductive region of samples with the same size was 160 (S3), 75 (S2), and 54 (S1). The contact point between stitches increased with the addition of tuck and float, and as a result, the total resistance of the conductive fabrics decreased with an increase in contact resistance. For double-needle-bed structures, since they consumed more yarn than single-needle-bed structures did, the resistance values were higher, relatively speaking. Among the three structures with double-needle beds, S4 had the smallest yarn consumption (232 cm) and the largest resistance (25.52 Ω). This was because the stitch number was higher in S5 (102) and S6 (120), which induced more contact points and higher contact resistance. The variation in different structures at 2 and 3 V was the same as the voltage at 1 V.

In terms of the change in resistance values under difference voltages, the resistance values decreased as the voltage values increased. When the voltage applied to the fabric increased, the current values measured by the Keithley sourcemeter also increased. According to Joule′s law, the heat generated by the current through the conductor is proportional to the current values. Therefore, the reason for the decrease in the resistance value measured by the increase in the applied voltages may have been due to the change in temperature. As the temperature increased, the resistance gradually decreased in the experiment.

### 4.2. Experiment 2 

**Figure 5:**
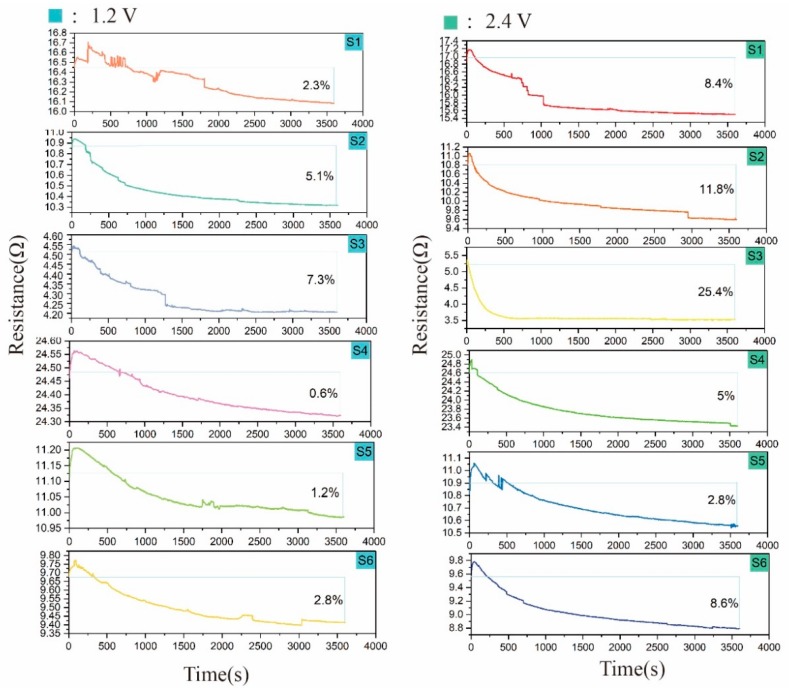
Resistance values of S1–S6 at 1.2 and 2.4 V for 1 h.

**Figure 6:**
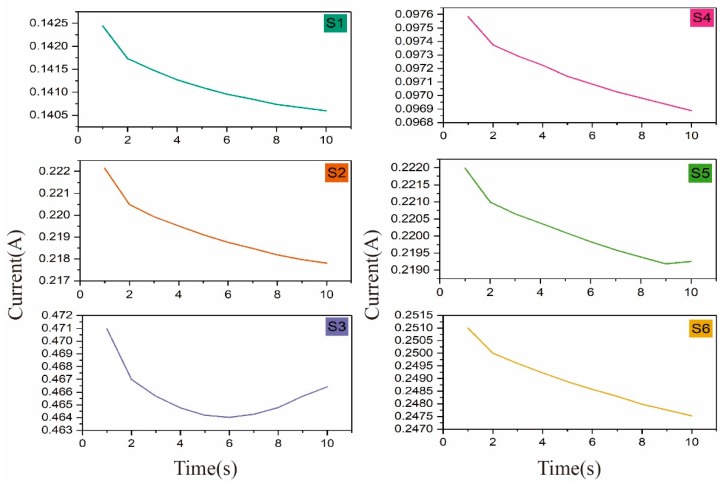
Current values of S1–S6 at 2.4 V in the first 10 s.

**Figure 7:**
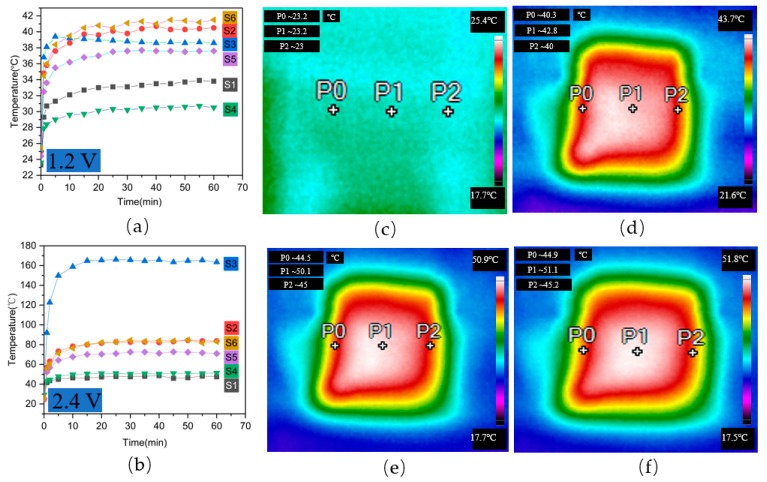
Sample temperature change with time of S1–S6 under (**a**) 1.2 V and (**b**) 2.4 V and thermal images of S4 (**c**) before and (**d**) after 1 min, (**e**) after 30 min, and (**f**) after 1 h of application of a voltage of 2.4 V.

The resistance values of the samples with six different structures and a conductive area with dimensions of 2 cm × 2 cm subjected to voltages of 1.2 and 2.4 V with time are shown in Figure 5. As can be seen from Figure 6, the current values of S1–S6 at 2.4 V in the first 10 s was reduced. It can be observed that the resistance rose briefly in the first few seconds and then decreased with time, and the percentage of resistance drop was more obvious at a voltage of 2.4 V (S1: 8.4%; S2: 11.8%; S3: 25.4%; S4: 5%; S5: 2.8%; and S6: 8.6%) compared to 1.2 V (S1: 2.3%; S2: 5.1%; S3: 7.3%; S4: 0.6%; S5: 1.2%; and S6: 2.8%). It can be concluded that the temperature had a greater influence on the resistance of the sample, and the higher the temperature was, the more obvious the drop was. When voltage was applied to the knitted conductive fabric, the heat generated in the conductive yarns raised the temperature of the fabric, and temperature affects the resistance value. This shows that the temperature values at 1.2 V for each structure (from high to low) were S6 (41 °C), S2 (40 °C), S3 (38 °C), S5 (37 °C), S1 (33 °C), and S4 (30 °C) (in Figure 7). However, it is not just that the higher the temperature was, the greater the magnitude of the resistance drop. The magnitude of the resistance drop was also related to the total number of stitches and the length of the conductive yarn in the conductive region. Among the structures, the temperature difference at 1.2 V of S6, S2, and S3 was within 3 °C, but since the total number of stitches of S3 (160) was the largest, the range of the resistance drop of S3 (7.3%) was the largest. In addition, the number of stitches of S6 (120) was greater than that of S2 (75), but the length of the conductive yarn of S6 (306 cm) was much larger than that of S2 (157 cm): the longer the length of the conductive yarn was, the smaller the range of resistance change after heating was (confirmed in Table 4), so the resistance change of S2 (5.1%) was greater than S6 (2.8%). For S5, S1, and S4, the temperature of S5 was the highest, but since the length of the conductive yarn of S5 (273 cm) was much larger than that of S1 (146 cm), the variation range of S5 (1.2%) was less than that of S1 (2.3%). The temperature of S4 was the lowest, and the range of resistance change was also the smallest. In general, temperature had the greatest effect on resistance change, followed by the length of the conductive yarn and the number of stitches in the conductive area. This rule was also established at a voltage of 2.4 V. 

In the first 10 s of applying voltage 2.4 V, the current of the sample was reduced. According to Joule′s law, the heat generation at the beginning is low, the heat generation of the sample is less than heat dissipation, and the temperature has little effect on the resistance. Therefore, the resistance has a short-term upward trend, and the tendency of resistance rise is higher at higher voltages. There are two main factors affecting the resistance of fabric: the length resistance and the contact resistance between the yarns. In order to confirm the effect of temperature on the length resistance, we heated yarn B at 80 °C for 15 min with three samples of different lengths. The resistance value before the yarn was heated and the resistance value after heating (to a cooling state) are shown in Table 4. After temperature treatment, the resistance of yarn B decreased. When the temperature rose, the conductive yarn and the wool yarn thermally expanded and the contact area between the conductive yarns increased, which caused the contact resistance of the fabric to increase. Combining the previous experimental results, an analysis can be drawn that the length resistance of the conductive yarn decreased, and the contact resistance between the yarns increased, so that the total resistance of the fabric decreased as the temperature rose under the influence of voltage. 

Figure 7 shows the temperature changes with time when voltages of 1.2 V (Figure 7a) and 2.4 V (Figure 7b) were applied to the samples and thermal images of 1 × 1 rib samples at 2.4 V. Under the effect of voltage, the temperature of the fabric gradually increased. In the early stage of the experiment, the temperature rose faster. At this time, the heat generation was greater than the heat dissipation. With an increase in time, the heat generation and heat dissipation of the fabric reached a balanced state, and the temperature of the fabric basically no longer changed. In the first 1000 s, the sample temperature rapidly increased with time and tended to stabilize for the remainder of the time, with applied voltages of 1.2 and 2.4 V. At 1.2 V, the temperature of S6 was the highest, reaching about 40 °C. At a voltage of 2.4 V, S3 had the highest temperature, reaching about 160 °C, but this temperature is too high to be used in heating textiles. In the process of heating, the resistance of the knitted fabrics with double-needle-bed structures changed little and was relatively stable, so this is more suitable for heating textiles. In addition, the temperature value (50 °C with S4 rib at a voltage of 2.4 V) was suitable for heating conductive textiles. The instrument used for the temperature test was an infrared thermal imager with three test points selected on each sample. The temperature of the sample before voltage treatment was about 23 °C, as shown in Figure 7c. Figure 7d–f indicates the temperature of the samples after 1 min (41 °C), 30 min (47 °C), and 1 h (47 °C) of heating. It can be seen that as the temperature increased, the area of the middle light-colored area gradually increased. 

**Figure 8:**
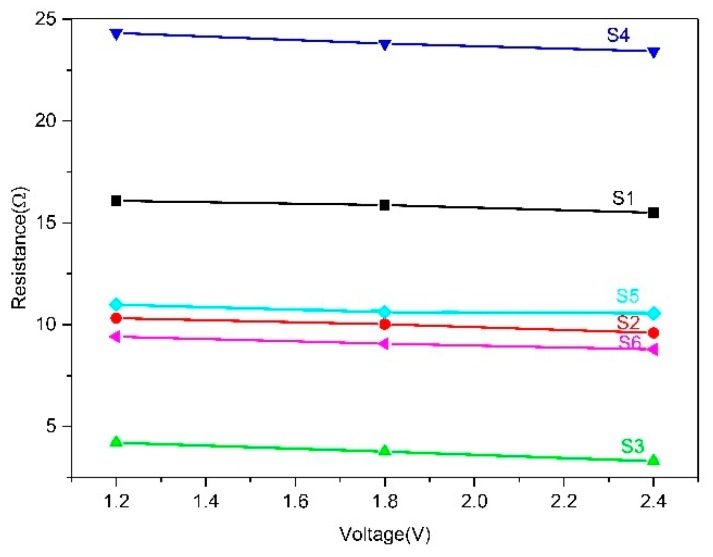
Sample resistance change with voltage of S1–S6 at 1.2, 1.8, and 2.4 V.

The resistance at voltages of 1.2, 1.8, and 2.4 V for different samples is plotted in Figure 8, and simulated equations of samples S1–S6 are shown in Table 5, where *R*_0_ is the resistance of S1, and *R* is the resistance of the other five structures in a steady state. The equation for the resistance versus voltage (*R*_0_–*U*) curve for S1 is Equation (7):

(7) $$R_{0}=16.69-0.49U.$$

Equation (6) becomes Equation (8):

(8) $$T_{s}{}^{\left(S1\right)}=24+\frac{U^{2}}{\alpha *\left(16.69-0.49U\right)}.$$

**Figure 9:**
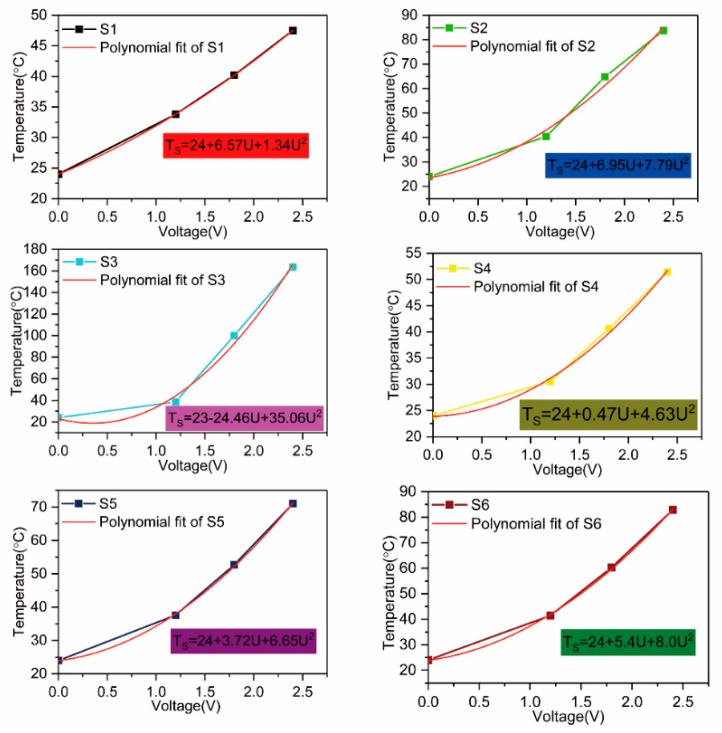
The experimental data and simulation data curves of temperature versus voltage (*T*–*U*) of S1–S6 at 1.2, 1.8, and 2.4 V.

Equation (8) is used to fit the temperature versus voltage (*T*–*U*) curve, and both the experimental data and simulation data curves are shown in Figure 9. The fitted heat dissipation coefficient is α = 0.01 (W/°C), and the error is within 0.001:

(9) $$T_{S}{}^{\left(S1\right)}=24+\frac{U^{2}}{0.1669-0.0049U}.$$

Using the same method, we found the equations for the temperature of the other fabric samples. 

According to the simulation equation, the κ value of different structures can be calculated: κ1:0.71 (S2); κ2:1.70 (S3); κ3:0.82 (S4); κ4:0.94 (S5); and κ5:0.85 (S6).

On the basis of the simulated voltage and resistance equations of samples S2–S6 in Table 5 (the κ values of different structures), the following equations are found:

(10) $$T_{s}{}^{\left(S2\right)}=24+\frac{U^{2}}{0.0364-0.0042U},$$

(11) $$T_{s}{}^{\left(S3\right)}=24+\frac{U^{2}}{0.0870-0.0129U},$$

(12) $$T_{s}{}^{\left(S4\right)}=24+\frac{U^{2}}{0.2066-0.0062U},$$

(13) $$T_{s}{}^{\left(S5\right)}=24+\frac{U^{2}}{0.1068-0.0034U},$$

(14) $$T_{s}{}^{\left(S6\right)}=24+\frac{U^{2}}{0.0852-0.0044U}.$$

Equations (9)–(14) can be used to predict the temperature of different structures for the entire range of voltage values.

## 5. Conclusions

This study, which examined the electrothermal properties of EKCFs with different structures subjected to different voltages, found that even though the electrothermal properties showed trends, they could be very different based on the differences in the knitted structure. Generally, the following conclusions are made:

(1) When power passed through the EKCFs, the resistance increased for a short period of time and then gradually decreased. The resistance increased a little in the first few seconds of the experiment, decreased fast within 1000 s, and then decreased slowly during the rest time. The temperature gradually increased in the first 1000 s and tended toward stability after a certain period of time;

(2) The factors affecting the electrical resistance of EKCFs were mainly related to the length resistance of the yarn and the contact resistance between the yarns. If the applied voltage was higher, the temperature increased, but an increase in temperature reduced the length resistance and contact resistance of the EKCFs. In terms of the experiments, S4 with a voltage of 2.4 V was most suitable for wearable conductive fabrics;

(3) Finally, the structural coefficient κ between different knitted structures and the thermal properties of different EKCFs subjected to different voltages were analyzed, and the findings can be used for further research work on thermal fabrics for wearable textiles.

## Figures and Tables

**Table 1:** Information on the specifications of the yarns in the samples.

Material	Yarn Count	Composition	Linear Resistance	Supplier
Silver-coated yarn A	47 tex	Nylon 66 coated with silver	1 Ω/cm	Germany Statex Produktions and Vertriebs GmbH
Silver-coated yarn B	2 tex	Nylon 6 coated with silver	69 Ω/cm	
Wool	77 tex	100% merino wool	Nonconductive	HongTai Cashmere Production Company
Polyester yarn	22 tex	Polyester fiber	Nonconductive	Tongxiang Tonglun Textile Co., Ltd.

**Table 2:** Knitting parameters of knitted samples.

Sample No.	Number of Transverse Knitting Stitches (pcs)	Number of Longitudinal Knitting Stitches (pcs)	Total Number of Stitches	NP Value (Related to Tension)	Pulling Value
S1	6	9	54	90	10
S2	5	15	75	82	10
S3	8	20	160	80	10
S4	10	10	100	70	10
S5	6	17	102	85	10
S6	6	20	120	85	10

**Table 3:** Resistance values (RVs) and standard deviations (SDs) of S1–S6 at 1, 2, and 3 V.

Voltage		1 V			2 V			3 V		
Sample No.		1	2	3	1	2	3	1	2	3
S1	RV	16.78	16.77	16.75	16.89	16.72	16.58	16.57	16.29	16.09
	*X* ± SD	16.77 ± 0.01			16.73 ± 0.13			16.32 ± 0.20		
S2	RV	11.79	11.79	11.7	11.85	11.73	11.48	10.97	10.6	10.38
	*X* ± SD	11.76 ± 0.04			11.69 ± 0.15			10.65 ± 0.24		
S3	RV	5.76	5.75	5.71	5.47	5.27	5.14	3.81	4.1	4.03
	*X* ± SD	5.74 ± 0.02			5.29 ± 0.14			3.98 ± 0.12		
S4	RV	25.71	25.54	25.3	25.38	25.3	25.27	25.4	25.23	25.07
	*X* ± SD	25.52 ± 0.17			25.32 ± 0.05			25.23 ± 0.13		
S5	RV	11.44	11.43	11.41	11.51	11.47	11.42	11.35	11.19	11.08
	*X* ± SD	11.43 ± 0.01			11.47 ± 0.04			11.21 ± 0.11		
S6	RV	10.36	10.34	10.32	10.39	10.29	10.20	10.02	9.75	9.55
	*X* ± SD	10.34 ± 0.02			10.29 ± 0.08			9.77 ± 0.19		

**Table 4:** Resistance change of yarn B at 80 °C.

Length (cm)	Resistance Before Heating (Ω)	Resistance After Heating (Ω)	Rate of Resistance Change (%)
4.5	290.45	241.83	−16.7
3.5	225.97	179.39	−20.6
3.2	210.45	154.86	−26.4

**Table 5:** Simulated voltage and resistance equations of samples S1–S6 at 1.2, 1.8, and 2.4V.

Sample No.	Equation
S1	*R*_0_ = 16.69 − 0.49*U*
S2	*R* = 11.06 − 0.60*U*
S3	*R* = 5.12 − 0.76*U*
S4	*R* = 25.20 − 0.76*U*
S5	*R* = 11.36 − 0.36*U*
S6	*R* = 10.02 − 0.52*U*

## References

[1] Stoppa M., Chiolerio A. (2014). Wearable Electronics and Smart Textiles: A Critical Review. Sensors.

[2] Lee J., Kwon H., Seo J., Shin S., Koo J.H., Pang C., Son S., Kim J.H., Jang Y.H., Kim D.E. (2015). Conductive Fiber-Based Ultrasensitive Textile Pressure Sensor for Wearable Electronics. Adv. Mater..

[3] Yi W. (2015). Flexible Fabric Strain Sensors.

[4] Atalay O., Kennon W. (2014). Knitted Strain Sensors: Impact of Design Parameters on Sensing Properties. Sensors.

[5] Seyedin S., Razal J.M., Innis P.C., Jeiranikhameneh A., Beirne S., Wallace G.G. (2015). Knitted Strain Sensor Textiles of Highly Conductive All Polymeric Fibers. Acs Appl. Mater. Interfaces.

[6] Castano L.M., Flatau A.B. (2014). Smart Fabric Sensors and E-textile Technologies: A Review. Smart Mater. Struct..

[7] Qureshi W. (2011). Integrating Conductive Threads into Different Knitting Construction by Flat Knitting Machine to Create Stretch Sensitive Fabrics for Breathing Monitoring. Ph.D. Thesis.

[8] King R.R., Miao X.H., Zhang S., Li Y.T., Wan A. (2018). Influence of Rib Structure and Elastic Yarn Type Variations on Textile Piezoresistive Strain Sensor Characteristics. Fibers Text. East. Eur..

[9] Yang M.Y., Pan J.J., Xu A.C., Luo L., Cheng D.S., Cai G.M., Wang J.F., Tang B., Wang X. (2018). Conductive Cotton Fabrics for Motion Sensing and Heating Applications. Polymers.

[10] Bhat N.V., Seshadri D.T., Nate M.M., Gore A.V. (2006). Development of Conductive Cotton Fabrics for Heating Devices. J. Appl. Polym. Sci..

[11] Bahadir S.K., Atalay O., Kalaoglu F., Vassiliadis S., Potirakis S. (2016). Performance Evaluation of Welded Knitted E-Fabrics for Electrical Resistance Heating. Proceedings of Second International Conference on Electrical Systems, Technology and Information 2015 (ICESTI 2015).

[12] Ehrmann A., Blachowicz T. (2017). Examination of Textiles with Mathematical and Physical Methods.

[13] Hao D., Xu B., Cai Z. (2018). Polypyrrole Coated Knitted Fabric for Robust Wearable Sensor and Heater. J. Mater. Sci..

[14] Ceken F., Kayacan Ö., Özkurt A., Uğurlu Ş.S. (2012). The Electromagnetic Shielding Properties of Some Conductive Knitted Fabrics Produced on Single or Double Needle Bed of a Flat Knitting Machine. J. Text. Inst..

[15] Tong J.H., Liu S., Yang C.X., Li L. (2015). Modeling of Package-free Flexible Conductive Fabric with Thermal Regulation where Temperature can be Customized. Text. Res. J..

[16] Liu S., Tong J.H., Yang C.X., Li L. (2017). Smart E-textile: Resistance Properties of Conductive Knitted Fabric—Single Pique. Text. Res. J..

[17] Liu S., Yang C.X., Zhao Y.F., Tao X.M., Tong J.H., Li L. (2016). The Impact of Float Stitches on the Resistance of Conductive Knitted Structures. Text. Res. J..

[18] Li L., Liu S., Ding F., Hua T., Au W.M., Wong K.S. (2012). Electromechanical Analysis of Length-related Resistance and Contact Resistance of Conductive Knitted Fabrics. Text. Res. J..

[19] Li L., Wai-man A., Ding F., Hua T., Wong K.S. (2014). Wearable Electronic Design: Electrothermal Properties of Conductive Knitted Fabrics. Text. Res. J..

[20] Tong J., Ding F., Tao X.M., Au W.M., Li L. (2014). Temperature Effect on the Conductivity of Knitted Fabrics Embedded with Conducting Yarns. Text. Res. J..

[21] Hamdani S.T.A., Fernando A., Hussain M.D., Potluri P. (2016). Study of Electro-thermal Properties of Pyrrole Polymerised Knitted Fabrics. J. Ind. Text..

